# Tolerance levels of mass density for CT number calibration in photon radiation therapy

**DOI:** 10.1002/acm2.12601

**Published:** 2019-05-13

**Authors:** Minoru Nakao, Shuichi Ozawa, Katsunori Yogo, Hideharu Miura, Kiyoshi Yamada, Fumika Hosono, Masahiro Hayata, Kentaro Miki, Takeo Nakashima, Yusuke Ochi, Daisuke Kawahara, Yoshiharu Morimoto, Toru Yoshizaki, Hiroshige Nozaki, Kosaku Habara, Yasushi Nagata

**Affiliations:** ^1^ Hiroshima High‐Precision Radiotherapy Cancer Center Hiroshima Japan; ^2^ Department of Radiation Oncology, Institute of Biomedical & Health Science Hiroshima University Hiroshima Japan; ^3^ Department of Radiology Nagoya University Graduate School of Medicine Nagoya Japan; ^4^ Radiation Therapy Section, Department of Clinical Support Hiroshima University Hospital Hiroshima Japan; ^5^ Department of Radiology Hiroshima Prefectural Hospital Hiroshima Japan; ^6^ Radiation Therapy Department Hiroshima City Hiroshima Citizens Hospital Hiroshima Japan; ^7^ Division of Radiology Hiroshima Red Cross Hospital & Atomic‐bomb Survivors Hospital Hiroshima Japan

**Keywords:** CT number calibration, mass density, tolerance level, radiation treatment planning, photon radiation therapy

## Abstract

Computed tomography (CT) data are required to calculate the dose distribution in a patient’s body. Generally, there are two CT number calibration methods for commercial radiotherapy treatment planning system (RTPS), namely CT number‐relative electron density calibration (CT‐RED calibration) and CT number‐mass density calibration (CT‐MD calibration). In a previous study, the tolerance levels of CT‐RED calibration were established for each tissue type. The tolerance levels were established when the relative dose error to local dose reached 2%. However, the tolerance levels of CT‐MD calibration are not established yet. We established the tolerance levels of CT‐MD calibration based on the tolerance levels of CT‐RED calibration. In order to convert mass density (MD) to relative electron density (RED), the conversion factors were determined with adult reference computational phantom data available in the International Commission on Radiological Protection publication 110 (ICRP‐110). In order to validate the practicability of the conversion factor, the relative dose error and the dose linearity were validated with multiple RTPSes and dose calculation algorithms for two groups, namely, CT‐RED calibration and CT‐MD calibration. The tolerance levels of CT‐MD calibration were determined from the tolerance levels of CT‐RED calibration with conversion factors. The converted RED from MD was compared with actual RED calculated from ICRP‐110. The conversion error was within ±0.01 for most standard organs. It was assumed that the conversion error was sufficiently small. The relative dose error difference for two groups was less than 0.3% for each tissue type. Therefore, the tolerance levels for CT‐MD calibration were determined from the tolerance levels of CT‐RED calibration with the conversion factors. The MD tolerance levels for lung, adipose/muscle, and cartilage/spongy‐bone corresponded to ±0.044, ±0.022, and ±0.045 g/cm^3^, respectively. The tolerance levels were useful in terms of approving the CT‐MD calibration table for clinical use.

## 1 Introduction

Computed tomography (CT) data are imported to a radiotherapy treatment planning system (RTPS), and it is required to structure a target and calculate the dose distribution in a patient’s body. In order to calculate a dose distribution in human body with RTPS, CT number calibration should be performed with several inserted tissue substitutes of a calibration phantom.^1^ Generally, there are two CT number calibration methods for photon radiation therapy, namely the CT number‐relative electron density calibration (CT‐RED calibration) and CT number‐mass density calibration (CT‐MD calibration). The CT number calibration methods are different based on each RTPS or dose calculation algorithm.

In a previous study,^2^ relative electron density (RED) tolerance levels were established for each tissue type. In the study, an effective depth was calculated with RED to water, and RED tolerance levels were determined with the effective depth and tissue maximum ratio (TMR). The tolerance levels of CT‐RED calibration are useful in terms of the quality assurance (QA) of the CT‐RED calibration table of planning CT and cone beam CT.^3^, ^4^ The tolerance levels of CT‐MD calibration are also useful for the QA because the CT‐MD calibration is used in several commercial RTPSes or dose calculation algorithms. However, the tolerance levels of CT‐MD calibration are not established yet. The tolerance levels are useful in approving the CT‐RED calibration table or CT‐MD calibration table for clinical use.

It is reasonable to determine the tolerance levels of CT‐MD calibration from the tolerance levels of CT‐RED calibration as opposed to determining the tolerance levels without any reference. The tolerance levels of CT‐RED calibration were determined by classifying standard tissues to five tissue groups including lung, adipose/muscle, cartilage/spongy‐bone, cortical bone, and tooth tissue.^5^ When the mass density (MD) is converted from RED within a tissue group, the conversion of pure organ is simple because MD and elemental composition are fixed. However, the conversion of multiple organs is complex because there are differences in the MD and elemental compositions between organs.

The purpose of this study involves determining the conversion factors from RED to MD with a whole body phantom and verifying the practicability of the conversion method. Furthermore, the tolerance levels for CT‐MD calibration are established in each tissue type.

## 2 Methods

### 2.1 Conversion factor between MD and RED in human body

**Figure 1:**
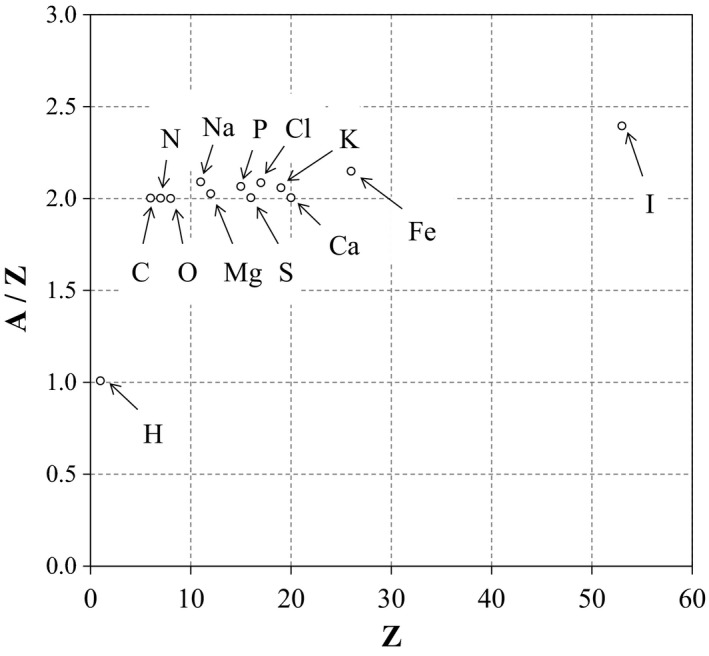
Ratio of mass number (A) to atomic number (Z) about human body component in ICRP‐110 phantom

The relationship between MD and electron density (ED) is given as follows:

(1) $$\rho_{e}={\rho N}_{A}\sum_{i}\frac{w_{i}Z_{i}}{A_{i}}$$

where *ρ*_e_ denotes ED; *ρ* denotes MD; *N*_A_ denotes the Avogadro’s number (6.022 × 10^23^); *i* denotes the element index; and *w_i_*, *Z_i_* and *A_i_* denote the weight, atomic number (Z), and atomic mass (A), respectively, of the *i*‐th element. The relationship between the atomic number (Z) and mass number (A) is different for each material. In order to determine the conversion factor for human body, we use the adult reference computational phantom data (V1.2) from the International Commission on Radiological Protection publication 110 (ICRP‐110).^6^ The reference anthropomorphic voxel phantoms are structured with 140 organs that consist of 52 standard tissues and air. The MD and elemental component of standard tissues are described for the complete body. Therefore, the voxel phantom is useful in determining the conversion factor between MD and RED. Based on ICRP‐110 phantom data, the natural human body consists of 13 elements. Figure 1 shows the ratio of mass number (A) to atomic number (Z) with respect to the 13 elements. The major materials under Z = 20, namely H, C, N, O, Na, Mg, P, S, Cl, K, and Ca, in which the sum of weight percent within whole body is larger than 99.9 wt%. On the other hand, the ratios of minor materials, which are Fe (A/Z = 2.2) and I (A/Z = 2.4), are higher than major material. However, the influence of minor material is negligible because the weight percent of minor materials smaller than 0.1 wt%. Thus, the ratio of material within whole body is approximately constant A/Z = 2 with the exception of hydrogen (A/Z = 1), and the conversion factors depend on the weight of hydrogen in tissue. Similar tissues are classified by MD into five tissue groups including lung, adipose/muscle, cartilage/spongy‐bone, cortical bone, and tooth tissues based on ICRP‐110 phantom data, and five conversion factors are determined for each tissue group.

The MD border between lung tissue and adipose tissue is ρ = 0.90 g/cm^3^.^7^ The MD of lung tissue is the only value below the border value of ρ = 0.90 g/cm^3^ while the MDs of adipose, muscle, general organ, and some spongy‐bone tissues are between 0.90 and 1.07 g/cm^3^. The MDs of skin, cartilage, and most spongy‐bone tissues are between 1.07 and 1.25 g/cm^3^. Furthermore, the MDs of cortical bone tissue and tooth tissue are 1.92 and 2.75 g/cm^3^, respectively.

Three tissue groups, namely the lung, cortical bone, and tooth, consist of one organ. However, adipose/muscle and cartilage/spongy‐bone consist of multiple organs. In order to determine averaged tissue with multiple organs, averaged MD and averaged ED are calculated with ICRU‐110 reference voxel phantom for adipose/muscle and cartilage/spongy‐bone. The averaged MD and averaged ED are given as follows:

(2) $$\overset{-}{\rho }=\frac{\sum_{j}\rho_{j}v_{j}}{V}\text{and}\overset{-}{\rho_{e}}=\frac{\sum_{ıj}{\left(\rho_{e}\right)}_{ıj}v_{ıj}}{V}$$

where $\overset{-}{\rho }$ and $\overset{-}{\rho_{e}}$ denote the averaged MD and averaged ED, respectively; *j* denotes the voxel index; *ρ*_*j*_, (*ρ*_e_)_*j*_, and *v*_*j*_ denote the MD, ED, and unit volume, respectively; of the *j*‐th voxel, and *V* denotes the sum volume of the tissue group. The conversion factors to MD form RED are given as follows:

(3) $$\overset{-}{\rho }=C\frac{\overset{-}{\rho_{e}}}{{\left(\rho_{e}\right)}_{H_{2}O}}$$

where C denotes the conversion factor to MD form RED. ${\left(\rho_{e}\right)}_{H_{2}O}$ denotes ED of water (${\left(\rho_{e}\right)}_{H_{2}O}$ = 3.34 ×10^23^). There are five conversion factors for five tissue groups. The conversion errors between actual RED of ICRP‐110 phantom data and RED converted from MD with the conversion factor are verified for adipose/muscle and cartilage/spongy‐bone.

### 2.2 Tolerance levels for CT‐MD calibration

The RED tolerance level based on TMR and effective tissue thickness were shown in previous study.^2^, ^5^ The RED tolerance levels were established to cause 2% dose error at effective tissue thickness with a 10 cm × 10 cm field. The tolerance levels are determined by the minimum usable energy in a RTPS because the tolerance levels are more strict with lower beam energy. The MD tolerance levels for photon beam are converted from RED tolerance levels. The conversion factors to MD from RED are determined based on ICRP‐110 phantom data for each tissue group. The MD tolerance levels are given as follows:

(4) $${\mathrm{TL}}_{MD}=C{\times TL}_{RED}$$

where *TL*_MD_ denotes the MD tolerance level, *TL*_RED_ denotes the RED tolerance level, and C denotes the conversion factor.

### 2.3 Practicability of the conversion factor

In order to validate the practicability of the conversion factors for MD tolerance levels in terms of eq. (4), the relative dose errors are compared between *TL*_MD_ and *TL*_RED_ for lung, adipose/muscle, and cartilage/spongy‐bone. Thus, the dose variation caused by MD variation is compared with that by RED variation. In other words, the relative dose error and the dose linearity are compared between CT‐MD calibration and CT‐RED calibration. However RTPS takes either CT‐MD calibration table or CT‐RED calibration table as input. The relative dose error and the dose linearity are validated with four RTPSes, namely Eclipse^TM^ planning system version 13.5 (Varian Medical Systems, Palo Alto, CA, USA), iPlan version 4.5 (BrainLAB, Feldkirchen, Germany), Pinnacle^3^ version 9.10 (Philips Healthcare, Andover, MA, USA), and Raystation version 6.2 (RaySearch Medical Laboratories AB, Stock‐holm, Sweden). The question of whether it is necessary to register the CT‐RED calibration or CT‐MD calibration differs based on the RTPS or dose calculation algorithm. The analytical anisotropic algorithm of Eclipse (Eclipse‐AAA) and pencil beam and Monte Carlo algorithms of iPlan (iPlan‐PC and iPlan‐MC) takes the CT‐RED calibration table as input. Conversely, the Acuros XB algorithm of Eclipse (Eclipse‐AXB), the collapsed cone convolution algorithms of Pinnacle^3^ and Raystation (Pinnacle^3^‐CCC and Raystation‐CCC) takes the CT‐MD calibration table as input. The MD range of the three tissue groups correspond to 0.25–0.41, 0.92–1.07, and 1.07–1.24 g/cm^3^ for lung, adipose/muscle, and cartilage/spongy‐bone, respectively. The RED are calculated from MD with conversion factors for each tissue group, and the converted CT‐RED calibration from CT‐MD calibration was used in Eclipse‐AAA, iPlan‐PC and iPlan‐MC. The averaged dose linearities for two groups, namely, the CT‐MD calibration and the converted CT‐RED calibration, are validated with multiple RTPSes. In particular, Eclipse‐AXB, Pinnacle^3^‐CCC and Raystation‐CCC are classified to the CT‐MD calibration group. Conversely, Eclipse‐AAA, iPlan‐PC and iPlan‐MC are classified to the converted CT‐RED calibration group. In order to compare the relative dose error and the dose linearity between four RTPSes or six dose calculation algorithms, a cubic region of interest (ROI), with a depth of 30 cm and larger than the irradiation field is formed with four RTPSes. The cubic ROI is filled with the MD of each tissue group or the RED converted from the MD with the conversion factor. The other dose calculation conditions are fixed through the following parameters: the linear accelerator corresponds to Varian TrueBeam STx (Varian Medical Systems, Palo Alto, CA, USA), photon beam energy corresponds to 6 MV, source‐to‐surface distance (SSD) corresponds to 90 cm, calculation depths correspond to 10 and 20 cm, the field size corresponds to 10 cm^2^ × 10 cm^2^ with a depth of 10 cm, the monitor unit (MU) is 500 MU, and the dose calculation grid is smaller than 3 mm. Thus, the absolute doses as a function of the same MD are compared between four RTPSes or six dose calculation algorithms. In order to decrease the difference of RTPS and dose calculation algorithm, the normalized dose was used to compare the relative dose error and the dose linearity. Normalized doses were calculated via dividing by the dose of reference MD. The reference MDs corresponded to 0.33, 1.00, and 1.16 g/cm^3^ for lung, adipose/muscle, and cartilage/spongy‐bone, respectively. The averaged dose linearity and the relative dose error for two groups are compared between the CT‐MD calibration group and the converted CT‐RED calibration group. The relative dose errors from the tolerance level of CT‐RED calibration or CT‐ MD calibration table are calculated as follows:

(5) $$D_{\text{err,}\;\text{RED}}={\mathrm{TL}}_{\text{MD}}\times P_{\text{RED}}\;\text{and}\;D_{\text{err,}\;\text{MD}}={\mathrm{TL}}_{\text{MD}}\times P_{\text{MD}}$$

where *D*_err,RED_ and *D*_err,MD_ denote the relative dose errors from the tolerance level of CT‐RED calibration and CT‐ MD calibration, respectively, *TL*_MD_ denotes the MD tolerance level, *P*_RED_ and *P*_MD_ denotes the proportionality factors of linear fitting curves for the CT‐MD calibration group and the converted CT‐RED calibration, respectively.

Furthermore the influence of the conversion factor on each tolerance level is validated as follows:

(6) $$\Delta D=D_{err,\;RED}-D_{err,\;MD}$$

where Δ*D* denotes the dose variation between CT‐RED calibration and CT‐ MD calibration.

## 3 Results

### 3.1 Conversion factors from MD to RED

Table 1 shows conversion factors from MD to RED by ICRP‐110 reference phantom. There were two reference phantoms in ICRP‐110 that included an adult male and adult female. Averaged conversion factors were collectively calculated from two reference phantoms. The values of male, the values of female, and average values were in agreement within 0.2%. Therefore, the average values were useful irrespective of gender.

**Table 1:** Conversion factors for lung, adipose/muscle, cartilage/spongy‐bone, cortical bone, and tooth

Classified tissue group	$\overset{-}{\rho }$(g/cm^3^)			$\overset{-}{\rho_{e}}$/${\left(\rho_{e}\right)}_{H_{2}O}$			*C* = $\overset{-}{\rho }\times {\left(\rho_{e}\right)}_{H_{2}O}$/ $\overset{-}{\rho_{e}}$ (g/cm^3^)		
	AM	AF	Average	AM	AF	Average	AM	AF	Average
Lung	0.385	0.385	0.385	0.382	0.382	0.382	1.009	1.009	1.009
Adipose/Muscle	1.012	0.998	1.006	1.006	0.994	1.001	1.006	1.004	1.005
Cartilage/Spongy‐bone	1.112	1.112	1.112	1.096	1.096	1.096	1.015	1.015	1.015
Cortical bone	1.92	1.92	1.92	1.784	1.784	1.784	1.076	1.076	1.076
Tooth	2.75	2.75	2.75	2.517	2.517	2.517	1.092	1.092	1.092

AM, adult male; AF, adult female.

**Figure 2:**
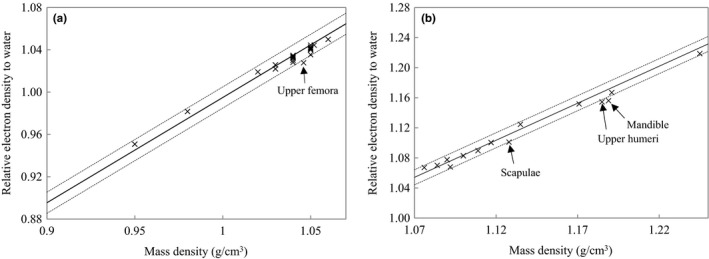
(a) and (b) show the relation between MD and RED to water for adipose/muscle and cartilage/spongy‐bone, respectively. The solid line denotes the converted RED from MD with the conversion factor. The dashed lines denote the converted RED ± 0.01. The plots (×) denote actual RED calculated with ICRP‐110 as a function of MD for each organ.

Figure 2 shows the relation between MD and RED to water for adipose/muscle and cartilage/spongy‐bone. The conversion factors of two tissue groups were determined from multiple organs. The converted RED from MD was compared with actual RED calculated with ICRP‐110. The difference was within ±0.01 for the most tissues. Conversely, the difference of upper femora, scapulae, upper humeri, and mandible were −0.013, −0.01, −0.013, and −0.015, respectively.

### 3.2 MD tolerance levels for tissue group

Table 2 shows the MD tolerance levels for each tissue group. The MD tolerance levels were estimated to cause 2% dose error at effective tissue thickness. The MD tolerance levels were calculated from RED tolerance levels with the conversion factors. The tolerance levels for cortical bone and tooth were not estimated because cortical bone and tooth were too thin to cause 2% dose error.^5^

**Table 2:** Summary of effective tissue thickness, RED tolerance level, conversion factor, MD tolerance level, *D*_err, RED_, *D*_err, MD_and Δ*D* for each tissue group

Classified tissue group	Effective tissue thickness (cm)	RED tolerance level	Conversion factor $\overset{-}{\rho }\times {\left(\rho_{e}\right)}_{H_{2}O}$/ $\overset{-}{\rho_{e}}$ (g/cm^3^)	MD tolerance level (g/cm^3^)	*D*_err,RED_	*D*_err,MD_	Δ*D*
Lung	10	±0.044	1.009	±0.044	−1.1%	−1.1%	<±0.1%
Adipose/Muscle	20	±0.022	1.005	±0.022	−1.5%	−1.4%	−0.1%
Cartilage/Spongy‐bone	10	±0.044	1.015	±0.045	−1.5%	−1.2%	−0.3%

The RED and MD tolerance levels are established to cause 2% dose error at effective tissue thickness. MD tolerance levels are converted from RED tolerance levels with conversion factors. *D*_err, RED_ and *D*_err, MD_ are defined in eq. (5). Δ*D* is defined in eq. (6).

MD, mass density; RED, relative electron density.

### 3.3 Practicability of the conversion factor

**Figure 3:**
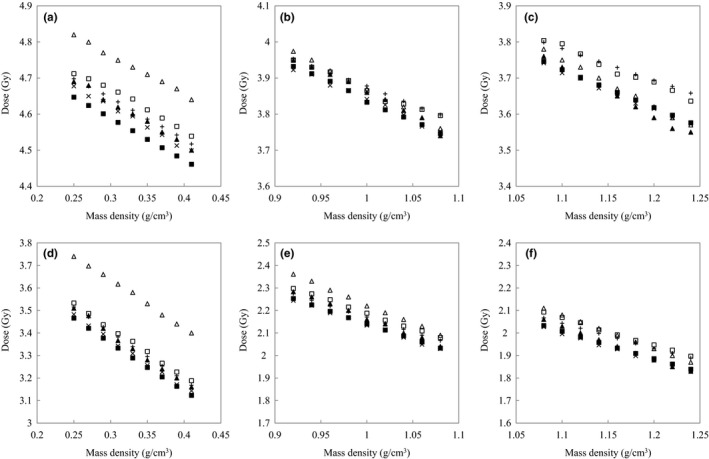
Absolute doses for each tissue group at depths of 10 cm or 20 cm. Figures (a), (b), and (c) are calculated at a depth of 10 cm. Figures (d), (e), and (f) are calculated at a depth of 20 cm. (a) and (d) correspond to the lung. (b) and (e) correspond to adipose/muscle. (c) and (f) correspond to cartilage/spongy‐bone. The absolute doses as a function of MD are compared between four RTPSes or six dose calculation algorithms including Eclipse‐AAA (□), Eclipse‐AXB (■), iPlan‐PC (△), iPlan‐MC (▲), Pinnacle3‐CCC (×), and Raystation‐CCC (＋).

Figure 3 shows the absolute doses compared with same MD between four RTPSes or six dose calculation algorithms for three tissue groups. The CT‐RED calibration converted from CT‐MD calibration is used for Eclipse‐AAA, iPlan‐PC and iPlan‐MC. The absolute doses decreased with increases in the MD.

**Figure 4:**
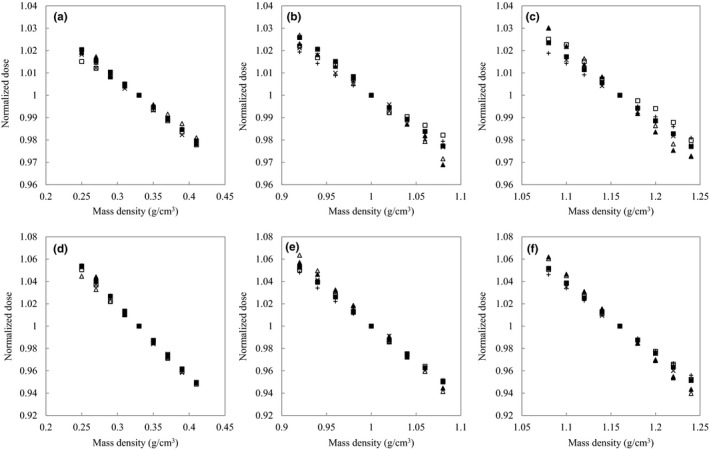
Normalized doses for each tissue group at depths of 10 cm or 20 cm. Figures (a), (b), and (c) are calculated at a depth of 10 cm. Figures (d), (e), and (f) are calculated at a depth of 20 cm depth. Figure (a) and (d) correspond to the lung. Figure (b) and (e) correspond to adipose/muscle. Figures (c) and (f) correspond to cartilage/spongy‐bone. The normalized doses as a function of MD are compared between four RTPSes or six dose calculation algorithms including Eclipse‐AAA(□), Eclipse‐AXB(■), iPlan‐PC(△), iPlan‐MC (▲), Pinnacle3‐CCC(×), and Raystation‐CCC(＋).

**Figure 5:**
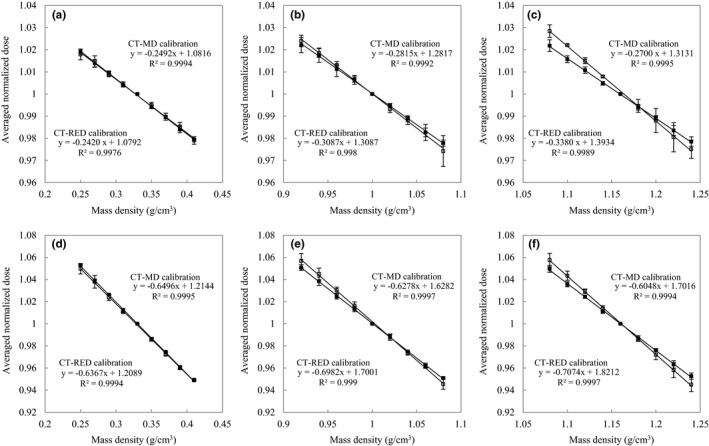
Averaged normalized doses and 1 SD for two groups, namely, the CT‐MD calibration (■) and the converted CT‐RED calibration (□) at depths of 10 cm or 20 cm. (a)–(c) are calculated at a depth of 10 cm. (d)–(f) are calculated at a depth of 20 cm. (a) and (d) correspond to the lung. (b) and (e) correspond to adipose/muscle. (c) and (f) correspond to cartilage/spongy‐bone.

With respect to the lung tissue group, the doses of iPlan‐PC exceeded the doses of iPlan‐MC although they used same CT‐RED calibration table. The relative dose difference between iPlan‐PC and iPlan‐MC at depths of 10 and 20 cm were 2.6%–3.1% and 6.3%–7.6%, respectively, for the lung tissue group. The coefficients of variation (CVs) were calculated with four RTPSes and six dose calculation algorithms for each tissue group. The ranges of CVs for the lung tissue group at depths of 10 and 20 cm were 1.2%–1.4% and 2.8%–3.2%, respectively. The ranges of CVs for adipose/muscle at depths of 10 and 20 cm were 0.4%–0.7% and 1.3%–1.8%, respectively. The ranges of CVs for cartilage/spongy‐bone at depths of 10 and 20 cm were 0.7%–1.3% and 0.5%–0.7%, respectively. Absolute doses were different based on the RTPS and dose calculation algorithm. Figure 4 shows each normalized doses for four RTPSes or six dose calculation algorithms. Figure 5 shows the averaged normalized doses and 1 standard deviation (SD) for two groups, namely, the CT‐MD calibration and the converted CT‐RED calibration. The effective tissue thicknesses, *D*_err, RED_, *D*_err, MD_ and Δ*D* for each tissue type are shown in Table 2.

## 4 Discussion

The goal of this study involved establishing MD tolerance levels of CT‐MD calibration for each tissue group. In a previous study,^2^, ^5^ the tolerance levels of CT‐RED calibration were determined by the effective tissue thicknesses and TMR data. However, the tolerance levels of CT‐MD calibration were not determined since the relationship between MD and effective tissue thickness was not established. In this study, tissue groups were determined by classifying with respect to MD, and conversion factors to MD from RED were created from standard whole body phantom data. The conversion error was within ±0.01 for most standard organs. The differences in several bone organs exceeded ±0.01 although it was assumed that the conversion error was sufficiently small because the thicknesses of the bone organs were thin and the conversion error was lower than the RED tolerance levels (Table 2). The ratio of atomic mass (A) to atomic number (Z) within major material (H, C, N, O, Na, Mg, P, S, Cl, K, and Ca) was approximately constant A/Z = 2 with the exception of hydrogen (A/Z = 1). Therefore, the conversion factor from RED to MD was affected by the weight of hydrogen based on eq.(1). We created five conversion factors by classifying all organs to five tissue groups based on MD.

In order to evaluate the practicability of the conversion factors when the tolerance levels of CT‐MD calibration were converted from the tolerance levels of CT‐RED calibration, the relative dose error and the dose linearity were compared between CT‐MD calibration and CT‐RED calibration for lung, adipose/muscle, and cartilage/spongy‐bone. However RTPS takes either CT‐MD calibration table or CT‐RED calibration table as input. Thus, the averaged dose linearity and the proportionality factors for two groups, namely, the CT‐MD calibration and the converted CT‐RED calibration, were validated with multiple RTPSes. Figure 3 shows the absolute doses as a function of the same MD for four RTPSes or six dose calculation algorithms. The differences in the absolute doses were affected by the RTPS and dose calculation algorithm. Specifically, the dose differences between iPlan‐PC and iPlan‐MC corresponded to 2.6%–3.1% at a depth of 10 cm for the lung tissue group although they calculated doses with same calibration table. The previous studies also indicated similar results.^8^, ^9^ The doses by normalizing the absolute dose at reference MD were compared for each tissue group in Fig. 4. The averaged dose linearities for two groups are compared between the CT‐MD calibration and the converted CT‐RED calibration in Fig. 5. *TL*_RED_ were established to cause 2% dose error with the effective depth and TMR measured in water. The relative dose errors caused by *TL*_RED_ were calculated in tissue material with RTPS, which were −1.1%, −1.5%, and −1.5% for lung, adipose/muscle, and cartilage/spongy‐bone, respectively. In addition, the relative dose errors caused by *TL*_MD_ were −1.1%, −1.4%, and −1.2% for lung, adipose/muscle, and cartilage/spongy‐bone, respectively. Thus, the relative dose errors calculated with RTPS were less than 2% for both of CT‐MD calibration group and the CT‐RED calibration group. The dose variations between *TL*_MD_ and *TL*_RED_ were less than 0.3% for lung, adipose/muscle, and cartilage/spongy‐bone, respectively, which were smaller than the relative doses errors caused by *TL*_RED_ or *TL*_MD_. One limitation in the current work is that the dose variation value was not validated with same dose calculation algorithm because RTPS takes either CT‐MD calibration table or CT‐RED calibration table as input. Instead of validating with same dose calculation algorithm, the practicability of the conversion factor and the tolerance levels for each tissue group were validated with multiple RTPSes and dose calculation algorithms.

The definition of tolerance levels of CT number calibration was useful for the QA of planning CT and cone beam CT.^3^, ^4^ It was important to define the tolerance levels with respect to the tissue type for dose calculations in inhomogeneous mediums because the accuracy of a dose calculation was stated as 2% for inhomogeneity.^10^ The CT number calibration table is generally obtained by using a calibration phantom with tissue substitutes. The CT number calibration may slightly vary for CT scan parameters or radiotherapy institutions due to the phantom size and amount of solid water around the density inserts. American Association of Physicists in Medicine Task Group 66 (AAPM TG‐66)^11^ has been the major standard for CT simulator quality control. In AAPM TG‐66, the tolerance level of CT number in Hounsfield units (HU) has been described for only water (0 ± 5 HU). Dose distribution in human body has been calculated with tissue materials, for example, lung, adipose muscle, and bone. However, the tolerance levels for each tissue type have not been mentioned in AAPM TG‐66. When clinical physicists register the CT number calibration table on the RTPS, the consistency is confirmed between all scan condition and CT number calibration table because CT number varies with scan conditions, which are tube voltage, reconstruction algorithm and acquisition field of view.^12^, ^13^ The tolerance levels were useful in approving the registered CT number calibration table in RTPS for clinical use.

## 5 Conclusion

In this study, we established the conversion factor from RED to MD with ICRP‐110 data. The RED and MD of organs were not proportional given the differences in the MD and element weights between organs. However, RED was converted to MD with a proportional conversion factor by classifying all organs into five tissues groups, and this study demonstrated the practicability of the conversion factors with four RTPSes and six dose calculation algorithms. Therefore, the tolerance levels for CT‐MD calibration were determined with conversion factors from the tolerance levels of the CT‐RED.

## CONFLICT OF INTEREST

The authors declare no conflict of interest.

## References

[1] Constantinou C , Harrington JC , DeWerd LA . An electron density calibration phantom for CT‐based treatment planning computers. Med Phys. 1992;19:325–327. 1584125 10.1118/1.596862

[2] Kilby W , Sage J , Rabett V . Tolerance levels for quality assurance of electron density values generated from CT in radiotherapy treatment planning. Phys Med Biol. 2002;47:1485–1492. 12043814 10.1088/0031-9155/47/9/304

[3] Richter A , Hu Q , Steglich D , et al. Investigation of the usability of conebeam CT data sets for dose calculation. Radiat Oncol. 2008;3:42. 19087250 10.1186/1748-717X-3-42 PMC2648965

[4] de Smet M , Schuring D , Nijsten S , Verhaegen F . Accuracy of dose calculations on kV cone beam CT images of lung cancer patients. Med Phys. 2016;43(11):5934–5941. 27806611 10.1118/1.4964455

[5] Nakao M , Ozawa S , Yamada K , et al. Tolerance levels of CT number to electron density table for photon beam in radiotherapy treatment planning system. J Appl Clin Med Phys. 2018;19:271–275. 10.1002/acm2.12226 PMC5768003 29152898

[6] Adult reference computational phantoms. ICRP Publication 110. Ann ICRP. 2009;39. http://www.icrp.org/publication.asp?xml:id=ICRP%20Publication%20110 10.1016/j.icrp.2009.09.001 19897132

[7] Kanematsu N , Inaniwa T , Nakao M . Modeling of body tissues for Monte Carlo simulation of radiotherapy treatments planned with conventional x‐ray CT systems. Phys Med Biol. 2016;61:5037–5050. 27300449 10.1088/0031-9155/61/13/5037

[8] Miura H , Masai N , Oh RJ , et al. Clinical introduction of Monte Carlo treatment planning for lung stereotactic body radiotherapy. J Appl Clin Med Phys. 2014;15(1):38–46. 10.1120/jacmp.v15i1.4202 PMC5711232 24423832

[9] Pokhrel D , Badkul R , Jiang H , Kumar P , Wang F . Technical note: Dosimetric evaluation of Monte Carlo algorithm in iPlan for stereotactic ablative body radiotherapy (SABR) for lung cancer patients using RTOG 0813 parameters. J Appl Clin Med Phys. 2015;16:349–359. 10.1120/jacmp.v16i1.5058 PMC5689968 25679161

[10] Papanikolaou N , Battista JJ , Boyer AL , et al. Tissue inhomogeneity corrections for megavoltage photon beams. AAPM Report No.85. Report of Task Group No. 65 of the Radiotherapy Committee. Madison, WI: Medical Physics Publishing 2004.

[11] Mutic S , Palta JR , Butker EK , et al. Quality assurance for computed‐tomography simulators and the computed‐tomography‐simulationprocess: report of the AAPM Radiation Therapy Committee TaskGroup no. 66. Med Phys. 2003;30:2762–2792. 14596315 10.1118/1.1609271

[12] Davis AT , Palmer AL , Pani S , Nisbet A . Assessment of the variation in CT scanner performance (image quality and Hounsfield units) with scan parameters, for image optimisation in radiotherapy treatment planning. Phys Med. 2018;45:59–64. 29472091 10.1016/j.ejmp.2017.11.036

[13] Davis AT , Palmer AL , Nisbet A . Can CT scan protocols used for radiotherapy treatment planning be adjusted to optimize image quality and patient dose? A systematic review. Br J Radiol. 2017;90:20160406. 28452568 10.1259/bjr.20160406 PMC5603945

